# Understanding Culture Clashes and Catalyzing Change: A Culture Cycle Approach

**DOI:** 10.3389/fpsyg.2019.00700

**Published:** 2019-04-11

**Authors:** Mar Yam G. Hamedani, Hazel Rose Markus

**Affiliations:** ^1^ Center for Social Psychological Answers to Real-World Questions (SPARQ), Stanford University, Stanford, CA, United States; ^2^ Department of Psychology, Stanford University, Stanford, CA, United States

**Keywords:** culture, multiculturalism, inequality, culture change, cultural divides, identity and conflict

## Abstract

U.S. Americans repeatedly invoke the role of “culture” today as they struggle to make sense of their increasingly diverse and divided worlds. Given the demographic changes, cultural interactions and hybridizations, and shifting power dynamics that many U.S. Americans confront every day, we ask how psychological scientists can leverage insights from cultural psychology to shed light on these issues. We propose that *the culture cycle*—a tool that represents culture as a multilayered, interacting, dynamic system of ideas, institutions, interactions, and individuals—can be useful to researchers and practitioners by: (1) revealing and explaining the psychological dynamics that underlie today’s significant culture clashes and (2) identifying ways to change or improve cultural practices and institutions to foster a more inclusive, equal, and effective multicultural society.

U.S. Americans are calling out the role of “culture” today as they struggle to make sense of their increasingly diverse and divided worlds. To say “It’s cultural,” or “It’s a culture clash,” or “We need a culture change” is becoming idiomatic. People invoke culture as they confront pressing issues in business, government, law enforcement, entertainment, education, and more, and as they grapple with power and inequality in the institutions and practices of these domains (e.g., racism, sexism, classism, homophobia, imperialism). Headlines and social media feeds are populated daily with news of culture clashes or cultural divides that take place both within organizations and across society. From gender clashes between men and women in the workplace, to race clashes between the police and communities of color in American suburbs and cities, to political clashes between conservatives and liberals around the nation, cultural differences and cultural misunderstandings are consistently in the spotlight (Armacost, 2016; Vance, 2016; Chang, 2018).

At the heart of these culture clashes are questions about the meaning and nature of social group differences, as well as the ways in which these differences are more often than not constructed as forms of inequality and marginalization (Markus, 2008; Markus and Moya, 2010; Salter and Adams, 2013; Adams et al., 2015; Omi and Winant, 2015; Adler and Aycan, 2018). Given the demographic changes, cultural interactions and hybridizations, and shifting power dynamics that many U.S. Americans confront every day, we ask how psychological scientists can leverage insights from cultural psychology to shed light on these issues. We propose that *the culture cycle*—a schematic or tool that represents culture as a multilayered, interacting, dynamic system of ideas, institutions, interactions, and individuals—can be useful to researchers and practitioners by: (1) revealing and explaining the psychological dynamics that underlie today’s significant culture clashes and (2) identifying ways to change or improve cultural practices and institutions to foster a more inclusive, equal, and effective multicultural society.

## The Culture Cycle

When psychological scientists theorize about the role of culture, the focus is often on how psychological processes are implicitly and explicitly shaped by features of the sociocultural contexts or worlds that people inhabit, as well as how these psychological processes in turn reflect and reproduce those sociocultural contexts or worlds (Markus and Conner, 2014; Gelfand and Kashima, 2016; Cohen and Kitayama, 2019). Psychologists Morris et al. (2015), for example, define culture as “a loosely integrated system of ideas, practices, and social institutions that enable coordination of behavior in a population” (p. 632). Other scholars (e.g., Shweder, 1991, 2003; Adams and Markus, 2004), drawing on the insights of anthropologists Kroeber and Kluckhohn (1952), expand on this idea and also highlight the dynamic, ongoing processes by which “the cultural” and “the psychological” necessarily and mutually depend upon as well as co-construct one another:

Culture consists of explicit and implicit *patterns* of historically-derived and selected *ideas* and their embodiment in *institutions, practices,* and *artifacts*; cultural patterns may, on one hand, be considered as products of action, and on the other as conditioning elements of further action. (as summarized by Adams and Markus, 2004, p. 341)

**Figure 1:**
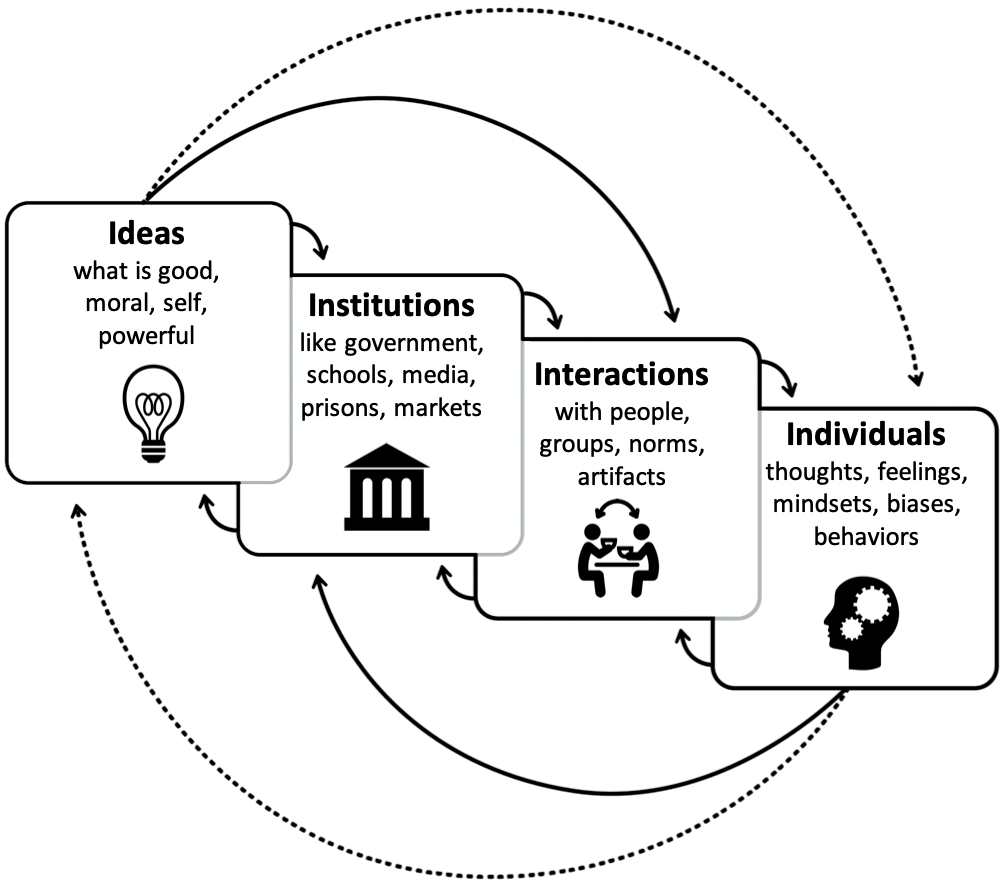
The culture cycle. Adapted from Fiske et al. (1998), Markus and Conner (2014), and Markus and Kitayama (2010).

This definition conceptualizes culture as a system or a cycle. In this cycle, sociocultural patterns shape or guide people’s actions, while people’s actions, in turn, can either reinforce and reflect or contest and change these sociocultural patterns. To visually and conceptually represent the dynamic processes through which the cultural and the psychological interact and mutually constitute one another, we use a tool that we call the “culture cycle” (Figure 1). This schematic depicts culture as a system of four, dynamically interacting and interdependent layers (Fiske et al., 1998; Markus and Kitayama, 2010; Markus and Conner, 2014). Here, culture is made up of the ideas, institutions, and interactions that guide and reflect individuals’ thoughts, feelings, and actions (Markus and Conner, 2014).

Analytically, the culture cycle starts from either the left-hand or the right-hand side. From the left, the ideas, institutions, and interactions of an individual’s mix of cultures shape the self, so that a person thinks, feels, and acts in ways that reflect and perpetuate these cultures. From the right, individuals participate in and create (i.e., reinforce, resist, and/or change) cultures to which other people, both in the present and throughout time, adapt. Psychologists typically focus on the *individuals level*, which includes identities, self-concepts, thoughts, feelings, mindsets, biases, and behaviors. These psychological processes can be culturally shaped as well as feed back into the cycle to shape culture (e.g., Markus and Kitayama, 2010; Varnum et al., 2010; Boiger and Mesquita, 2012).

The next layer of the culture cycle is the *interactions level*. As people interact with other people and with human-made products (i.e., cultural artifacts), their ways of life manifest in everyday situations that follow seldom-spoken norms about the right ways to behave at home, school, work, worship, and play. Guiding these practices are the everyday cultural products—the stories, songs, advertisements, social media, and tools (e.g., phones, laptops, tablets)—that make some ways to think, feel, and act easier, more fluid, or better supported by the particular worlds a person inhabits (e.g., Tsai et al., 2007; Morling and Lamoreaux, 2008; Lamoreaux and Morling, 2012).

The next layer of culture is made up of the *institutions level*, within which everyday interactions take place. Institutions spell out and formalize the rules for a society and include government, religious, legal, economic, educational, and scientific institutions. For the most part, people may be unaware of all the institutions, laws, and policies at play in their cultures. Yet they exert a formidable force by providing incentives that foster certain practices, interactions, and behaviors while inhibiting others (e.g., Hatzenbuehler et al., 2010, 2014; Tankard and Paluck, 2017).

The last layer of the culture cycle is the *ideas level*, and it is made up of the pervasive, often invisible, historically derived and collectively held ideologies, beliefs, and values about what is good, right, moral, natural, powerful, real, and necessary that inform institutions, interactions, and ultimately, individuals (e.g., Hamedani et al., 2013; Leavitt et al., 2015; Master et al., 2016). Because of them, cultures can appear to have overarching themes or patterns that persist, to some extent, across time. To be sure, cultures have multiple exceptions to their own foundational rules and values. But they also contain general patterns that can be detected, studied, and changed.

A few clarifying notes on the culture cycle. First, all four interacting layers of the culture cycle are important and mutually depend upon one another; none is assumed to be more influential, theoretically prior to, or separable from the others. Second, cultures are always dynamic, never static, and can change or evolve over time. As such, all levels continually influence each other and a change at any one level can produce changes in other levels. Third, the culture cycle includes structures and structural dynamics and does not separate the concept of “culture” from “structure.” And finally, culture cycles are embedded within larger natural and ecological systems that can interact with and exert influence on a given culture.

Many different kinds of cultures can be mapped and analyzed using the culture cycle (Cohen, 2014; Markus and Conner, 2014; Gelfand and Kashima, 2016; Cohen and Kitayama, 2019). Culture can be geographically based and focus on familiar distinctions—such as the East versus the West or the Global North versus the Global South—but it also encompasses other distinctions like social class or socioeconomic status; race, ethnicity, or tribe; gender and sexuality; region of the country, state, or city; religion; profession, workplace, or organization; generation; or immigration status. “Culture” or “cultural context” can serve as a label for any significant (i.e., socially meaningful) category associated with a set of shared ideas, practices, and products that structure and organize behavior. Since the cultural and the psychological make each other up, one way to change minds and behaviors is to change cultures, just as one way to change cultures is to change minds and behaviors.^1^

## Using the Culture Cycle to Understand Culture Clashes and Catalyze Change

**Figure 2:**
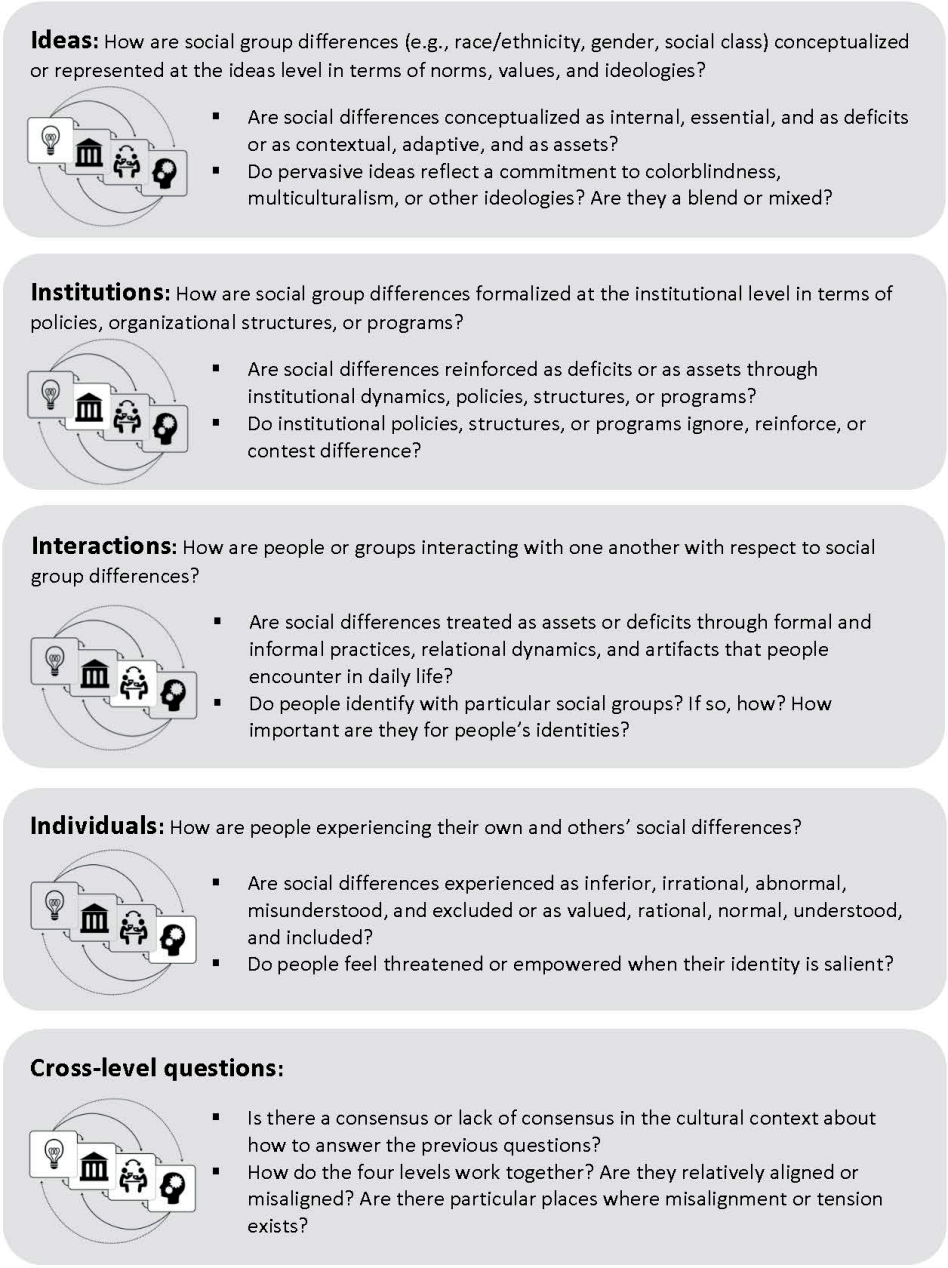
Using the culture cycle to understand culture clashes and catalyze change: Mapping social group differences. Adapted from Markus and Hamedani (2019).

We propose that addressing current culture clashes and divides through more inclusive, equal, and effective institutions and practices will require changing how people encounter and experience the meaning and nature of social group differences themselves (Markus, 2008; Markus and Moya, 2010; Plaut, 2010). At the heart of today’s most timely culture clashes and divides is a pervasive process of devaluing the less powerful or non-dominant group in contrast with the more powerful or dominant group. In the process, differences are cast as the result of so-called negative and inherent shared behavioral characteristics or tendencies rather than as a matter of divergent life experiences or differential access to resources, power, and/or status—e.g., women = incompetent (versus men = competent), black = criminal (versus white = lawful), and liberals = weak (versus conservatives = strong; e.g., Prentice and Carranza, 2002; Eberhardt et al., 2004; Graham et al., 2012). To analyze how cultural differences are constructed and understood in a given setting, we recommend starting with the following set of orienting questions (Figure 2). These questions are designed to help prospective culture changers map how social differences are constructed within a given culture cycle (e.g., as assets versus deficits, through colorblind versus multicultural ideologies), identify where inequalities exist (e.g., at the ideas, institutions, interactions, and/or individuals levels), and locate places within the culture cycle to intervene. To provide an example, we apply this method to unpack the cultural and psychological dynamics that underlie one culture clash prevalent on U.S. American college campuses today—the clash between underrepresented students (e.g., low-income students and/or students of color) and the mainstream (e.g., middle- to upper-class and White) culture of higher education (Wong, 2015; Wong and Green, 2016).

The culture of American higher education, especially at elite colleges and universities, reflects and promotes assumptions about what it means to be “smart,” “educated,” and “successful.” These assumptions are not neutral, but are instead powerfully shaped by White, middle- to upper-class beliefs, norms, and values that privilege independence and innate intelligence (Fryberg et al., 2013; Quaye and Harper, 2014; Canning et al., 2019). As a result, students of color and students from low-income or working-class backgrounds often feel excluded in these educational settings due to threats to their social identities (e.g., stereotypes about race and intelligence) or mismatches with their interdependent norms and values (e.g., achieving for one’s family or community instead of oneself; Ostrove and Long, 2007; Walton and Cohen, 2007; Stephens et al., 2012; Covarrubias and Fryberg, 2015). These experiences of exclusion can lead students to question whether they fit or belong in college. Students from low-income or working-class backgrounds can also be unfamiliar with the “rules of the game” needed to succeed in higher education, which can undermine their sense of empowerment and efficacy (Housel and Harvey, 2010; Reay et al., 2009). These psychological challenges work alongside disparities in resources and pre-college preparation to fuel a persistent achievement gap between these students and their advantaged peers (Astin and Oseguera, 2004; Bowen et al., 2005; Sirin, 2005; Goudeau and Croizet, 2017). As such, the culture clash that results from participating in mainstream college environments can systematically disadvantage underrepresented students (Stephens et al., 2012; Brannon et al., 2015; Covarrubias et al., 2016).

What kinds of culture clashes do underrepresented students experience at each level of a college or university’s culture cycle? Where might practitioners intervene to make a college or university’s values, policies, and practices more inclusive and equitable? Using the orienting questions in Figure 2, we can map this culture clash as well as corresponding interventions at each layer or level of the cycle. Starting with the *individuals level* (Figure 2*: How are people experiencing their own or others’ social differences?*), research shows that underrepresented students often feel like they do not fit or belong on college and university campuses, which can be due to repeated everyday experiences like microaggressions that take place at the *interactions level* (Figure 2*: How are people or groups interacting with one another with respect to social group differences?*) during intergroup encounters in classrooms or in the dorms (Yosso et al., 2009; Sue, 2010). These factors can lead students to experience the college environment as threatening to their social identities and to view their social differences as deficits or as something that puts them at a disadvantage.

At the *institutions level* (Figure 2*: How are social group differences formalized at the institutional level in terms of policies, organizational structures, or programs?*), these threats to fit or belonging can be reinforced in multiple ways, including a lack of representation in the college curriculum (e.g., not seeing people with your background reflected in lecture examples, readings, and research), and in positions of authority throughout the university (e.g., as faculty and administrators; Brannon et al., 2015; Quaye and Harper, 2014). Further, at the *ideas level* [Figure 2*: How are social group differences* (*e.g., race/ethnicity, gender, social class*) *conceptualized or represented at the ideas level in terms of norms, values, and ideologies?*], while many college and universities today claim to value diversity, they rarely do so in ways that include and affirm underrepresented students’ backgrounds and experiences—that challenge prevailing assumptions about what it means to be a smart, educated, or successful student (i.e., an independent and innately intelligent student; Chang, 2002; Stephens et al., 2012). Underrepresented students’ backgrounds and ways of being, therefore, are frequently devalued or seen as deficits in mainstream colleges and universities rather than valued and seen as assets or resources, which undermines such commitments to diversity, equity, and inclusion and reinforces color- or identity-blindness.

To change their cultures, colleges and universities need to do more to challenge how students’ social differences are experienced and constructed at each layer of the culture cycle. Research suggests several evidence-based strategies to catalyze culture change and make higher education more inclusive and equitable. To help underrepresented students feel more included and empowered at the *individuals level*, for instance, colleges and universities can do more to value and promote diversity, equity, and inclusion as crucial components of a high-quality education for a twenty-first century workforce in their missions and institutional strategies at the *ideas level* (Hurtado, 2007; Gurin et al., 2013). Next, at the *institutions level*, colleges and universities can integrate intergroup dialogue classes and other learning experiences about diversity, equity, and inclusion into the college curriculum for all students and across all courses of study, as well as implement hiring and promotion policies that foster the diversification of faculty and administrators (Hurtado, 2005; Gurin et al., 2013; Brannon, 2018; Stephens et al., 2019). At the *interactions level*, colleges and universities can better support students by providing opportunities for them to expand their networks and connect with mentors and alumni that share their backgrounds and have found pathways to success (Girves et al., 2005; Harper, 2008). While some of these strategies focus on transforming the norms of higher education itself, others involve better supporting students on their journeys through institutions that still have much work to do. None of these changes alone are a panacea, and may fail to support long-term and sustainable change if they are not built into and fostered by the larger college culture as well as lived out and reinforced through the everyday actions of the people in that culture.

Ideally, culture change is most likely to progress and have the greatest impact when there is change at each level of the culture cycle and these changes work together to support one another. As noted previously, all four levels of the culture cycle are equally influential. When it comes to culture change, however, culture changers need to consider whether the levels are working together to reinforce or buttress one another, or whether they might be working against one another, causing spots of tension and misalignment in a culture (Porras and Silvers, 1991; Morgan, 2006; Kotter, 2012; Gibbons, 2015; Coyle, 2018; see the Figure 2 “cross-level” questions). For example, if colleges and universities express a commitment to diversity, equity, and inclusion at the *ideas level*, but fail to take a hard look at how their current policies, programs, and practices are impacting underrepresented students at the *institutions* and *interactions levels*, diversity efforts are likely to be seen as disingenuous by student communities and culture change efforts are likely to have a limited influence on the institution as a whole.

Prospective culture changers also need to consider whether people within a given cultural context have consensus or a shared understanding of what is taking place and why in a given setting (see also the Figure 2 “cross-level” questions). For example, students from underrepresented groups and administrators at colleges and universities (many of whom are from majority groups) may have divergent perspectives on how to make change in their institutions with respect to diversity, equity, and inclusion. Students may favor more bottom-up, transformative efforts that are instigated by their peers, while administrators might favor more top-down, incremental changes brought about from long-term institutional study. While both groups might have valid perspectives, they might buy into and trust different culture change strategies. Culture change efforts that ignore the ideas and strategies of the lower status or low power side of the clash, however, are likely to be less effective than those that incorporate them.

## Concluding Comments

The phrase “It’s cultural” underscores the frustration that people feel when a problem is big, messy, and seems intractable. Sometimes people use it as a way to say that a problem is systemic, but they also often use it as a way to evade responsibility and say that a significant societal problem is not really their problem. We do not deny that culture change is difficult work and may have unintended consequences. Culture changers need to keep in mind how the interconnected, shifting dynamics that make up the culture cycle afford certain ways of being while constraining or downwardly constituting others, and that these dynamics can change or rebalance when intervening in the cycle. Culture changers also need to recognize that to foster more inclusive, equal, and effective institutions and practices, the deeper work will involve changing how cultures construct the meaning and nature of social group differences themselves.

Given that psychologists are typically trained to focus on the individual and sometimes the interactional levels, they tend to zero in on changing people’s mindsets or construals without fully considering how these micro- or meso-level changes might be blocked rather than supported by the larger institutional and social forces at play. On the other hand, practitioners and policymakers often focus on macro-level social and institutional factors and, in turn, do not pay close enough attention to whether the changes have resonance and carry over to the interactional and individual levels. Both psychologists and practitioners alike can also overlook the power individuals have to change their cultures in bottom-up ways through their actions, by instead focusing on how cultures shape people rather than how people also shape their cultures. With these considerations in mind, a culture cycle approach can be useful to scholars and practitioners alike to help them anticipate areas of misalignment and tension, forecast unanticipated consequences, and foster more holistic, dynamic, and multidirectional approaches to culture change.

## Author Contributions

Both authors contributed to the theory, conceptualization, and writing of the paper. MH had primary responsibility for writing the manuscript.

### Conflict of Interest Statement

The authors declare that the research was conducted in the absence of any commercial or financial relationships that could be construed as a potential conflict of interest.

## Footnotes

^1^The kind of intentional or strategic culture change that we discuss here differs from other significant work in the field on cultural evolution or long-term social change, which is primarily concerned with demonstrating and documenting how cultures or societies shift, change, or evolve across time (e.g., Twenge et al., 2012; Greenfield, 2013; Varnum and Grossmann, 2017).
